# Expression of long non-coding RNA DLX6-AS1 in lung adenocarcinoma

**DOI:** 10.1186/s12935-015-0201-5

**Published:** 2015-05-02

**Authors:** Juan Li, Ping Li, Wei Zhao, Rui Yang, Shanshan Chen, Yong Bai, Shaozhi Dun, Xiaonan Chen, Yuwen Du, Yuanyuan Wang, Wenqiao Zang, Guoqiang Zhao, Guojun Zhang

**Affiliations:** Department of Respiratory Medicine, The First Affiliated Hospital of Zhengzhou University, Zhengzhou, Henan China; Department of Pediatric Surgery, The First Affiliated Hospital of Zhengzhou University, Zhengzhou, Henan China; College of Basic Medical Sciences, Zhengzhou University, Zhengzhou, Henan China

**Keywords:** lncRNA DLX6-AS1, LAC, Microarray assay, DLX6

## Abstract

**Background:**

Lung adenocarcinoma (LAC), the primary histological type of non-small cell lung cancer (NSCLC), has displayed an increasing incidence and mortality worldwide. However, therapeutic approaches were limited. Dysregulation of some lncRNAs has been shown in various types of cancers including LAC. The aim of the present study was to vertify lncRNA DLX6-AS1 expression in LAC.

**Methods:**

Microarray assay revealed expression profile of lncRNAs in LAC. qRT-PCR ( quantitative reverse transcription PCR) was performed to identify lncRNA DLX6-AS1 expression level in 72 paired LAC and adjacent normal lung tissues. qRT-PCR and Western blotting were used to verify that down-regulation lncRNA DLX6-AS1 decreased DLX6 (distal-less homeobox 6) mRNA and protein expression.

**Results:**

Microarray analysis identified up-regulation of 272 lncRNAs and down-regulation of 635 lncRNAs in LAC tissues. The expression level of lncRNA DLX6-AS1 in LAC tissues was significantly higher compared to paired adjacent normal lung tissues (*P*< 0.05). In addition, its expression level was closed correlated with both histological differentiation (*P* = 0.004) and TNM stage (*P* = 0.033). qRT-PCR and Western blotting analysis showed that DLX6 mRNA and protein levels were lower in si-LncRNA group than in the NC (negative control) and Blank groups.

**Conclusions:**

Microarray analysis identified that lncRNA DLX6-AS1 was up-regulated in LAC tissues. High DLX6-AS1 expression levels were significantly associated with both histological differentiation and TNM stage. Down-regulation of lncRNA DLX6-AS1 expression decreased the DLX6 mRNA and protein levels.

## Background

With an ascending incidence in recent year, lung cancer is now the mostly commonly diagnosed malignancy worldwide, representing the leading cause of cancer-related death [1-3]. In 2014, an estimated 224,210 new cases will be diagnosed, of which the majority are probably non-small cell lung cancer (NSCLC) and at advanced stage meanwhile [4]. NSCLC including adenocarcinoma and squamous cell carcinoma, is a predominant form of lung cancer [5]. Despite the improvement achieved in chemotherapy and radiotherapy over the past few decades, the prognosis for patients with NSCLC is still dismal, with 5-year survival rate slightly more than 15% [6]. Lung adenocarcinoma (LAC) accounts for more than 50% of all NSCLC and its incidence has been increasing recently [6,7]. Thus, it is urgent to find new prognostic markers and therapeutic strategies to improve the present situation. Recently, growing evidence indicates that non-coding RNAs may be involved in cancer pathogenesis, providing new insights into the biological progress of LAC [8,9].

The human genome project revealed that at least 90% of the human genome is actively transcribed to RNA, but less than 2% of RNA encodes proteins [10,11]. Non-coding RNAs (ncRNAs), including microRNAs (miRNAs), long non-coding RNAs (lncRNAs), tRNAs, snoRNAs, and siRNAs, are functional RNAs that don’t encode proteins [12,13]. LncRNAs are known to play important roles during cellular development and differentiation, and a large range of biological processes, such as modulation of tumor proliferation and invasiveness, and reprogramming of induced pluripotent stemcells [14,15]. Dysregulation of some lncRNAs has been shown in various diseases including cancers, such as breast cancer, hepatocellular carcinoma, melanoma, bladder cancer, prostate cancer, gastric cancer and skin cancer [16-27]. For example, HOTAIR, has been determined as a negative prognostic indicator in breast, liver and pancreatic cancer, representing a close association especially with breast cancer metastasis [28,29]. Takahashi’s study demonstrated that PVT1 expression levels in colorectal cancer tissues were significantly higher than that in non-cancerous tissue, and patients with high PVT1 expression had a significantly poorer prognosis, what’s more, knockdown PVT1 expression could promotes apoptosis in colorectal cancer cells [30]. Metastasis associated lung adenocarcinoma transcript 1 (MALAT1) was found to be over expressed not only in breast, pancreas, colon, prostate, and liver cancers, but also in early-stage metastasizing NSCLC [31,32].

However, the functions of lncRNAs in lung cancer remain unclear. We investigated microarray data of lncRNAs from 3 paired LAC tissues and adjacent normal tissues, then focused on lncRNA DLX6-AS1 (distal-less homeobox 6 antisense 1). Up to now, there is no relevant report about the relationship between lncRNA DLX6-AS1 and the progression of LAC. The aim of the present study was to vertify lncRNA DLX6-AS1 expression in LAC tissues and adjacent normal tissues, then to explore the relationship between lncRNA DLX6-AS1 expression and clinicopathological features and to test the expression of DLX6 mRNA and protein in LAC cell lines after transfection with siRNA of lncRNA DLX6-AS1. The study may provide a new perspective on the diagnosis and treatment for this deadly disease.

## Results

### Microarray data analysis

**Figure 1:**
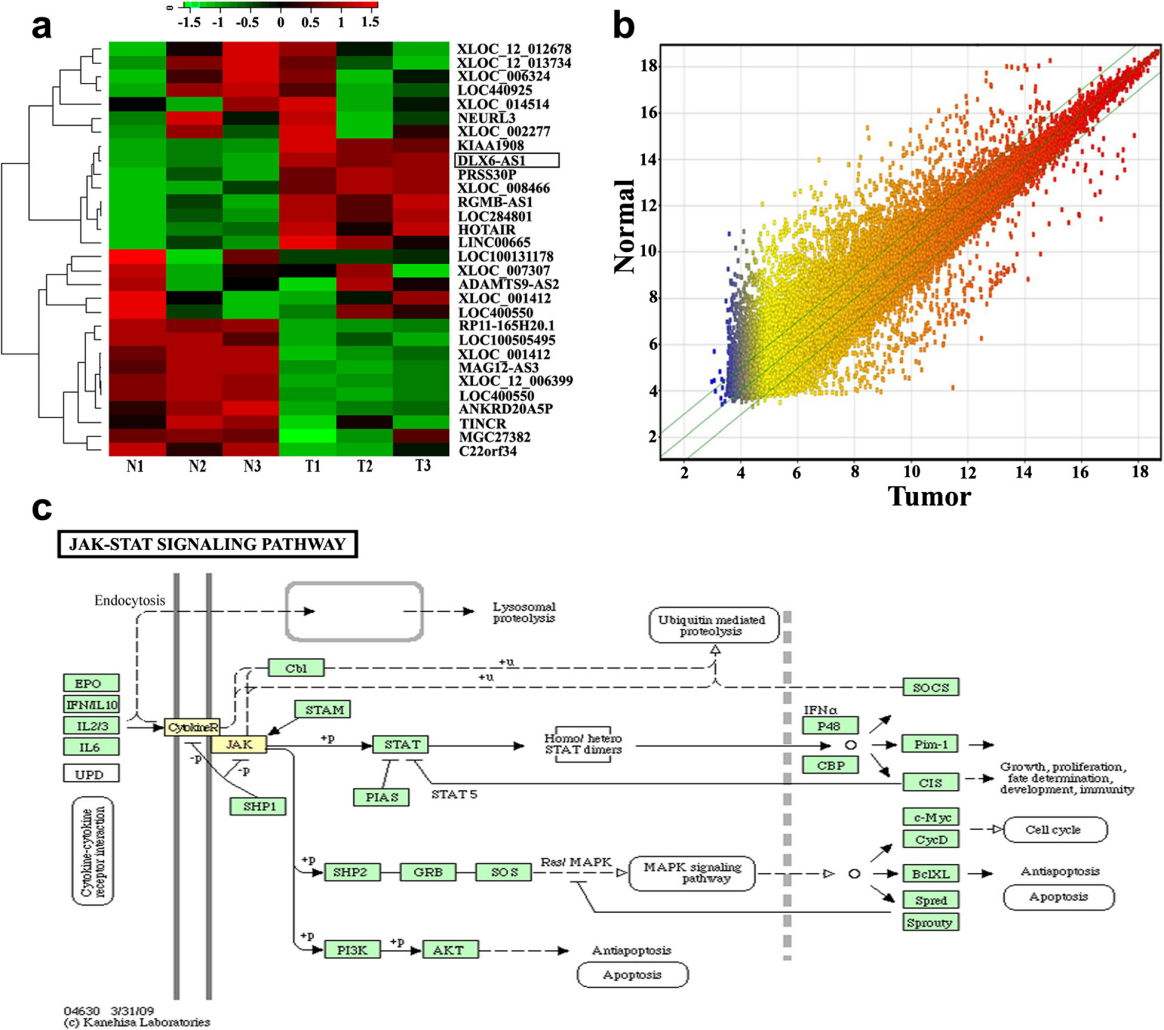
Microarray data analysis. **a** The result from Hierarchical Clustering showed part of the distinguishable lncRNA expression profiles among 3 paired LAC and adjacent normal tissues. “Red” indicates high relative expression; and “green” indicates low relative expression. **b** The scatter-plot was used for assessing the lncRNA expression variation among 3 paired LAC and adjacent normal tissues. The values of x-axis and y-axis in the scatter-plot were the normalized signal values of each sample (log 2 scaled). The green lines are fold change lines (the default fold change value given is 2.0). The lncRNAs above the top green line and below the bottom green line indicated > 2.0-fold change in expression of lncRNAs among the 3 compared samples. “Red” denotes high relative expression levels, and “blue” denotes low relative expression levels. **c** KEGG pathway analysis revealed that lncRNA DLX6-AS1was involved in cell proliferation process with JAK/STAT signaling pathway. The yellow squares represent key elements in cell proliferation progression.

A total of 907 lncRNAs were demonstrated with differential expressions (Log_2_fold change (T/N ≥2 or ≤ −2) between tumor tissues and adjacent normal tissues from 3 LAC patients. Microarray analysis identified that 272 lncRNAs were up-regulated including lncRNA DLX6-AS1, whereas 635 lncRNAs were down-regulated in LAC tissues. Part of lncRNAs expression levels are shown in Table 1. The heat map of the hierarchical clustering results was performed to vertify part of the distinguishable lncRNAs expression profile among 3 paired LAC and adjacent normal tissues (Figure 1a). The scatterplot results showed that the distribution and expression variation of the log 2 ratios of lncRNAs were similar among 3 paired LAC and adjacent normal tissues (Figure 1b). KEGG pathway analysis revealed that lncRNA DLX6-AS1was involved in cell proliferation process through JAK/STAT signaling pathway (Figure 1c). The molecular mechanism will be clarified in our further study.

**Table 1:** **Part of lncRNAs detected using microarray in three LAC patients**

**Upregulated in cancer**		**Downregulated in cancer**	
**lncRNAs**	**Log**_**2**_ **fold change (T/N)**	**lncRNAs**	**Log**_**2**_ **fold change (T/N)**
LOC145757	4.1850148	ANKRD20A5P	-2.416844
DLX6-AS1	3.893559	C22orf34	-2.4658422
LOC284801	3.6185247	MAGI2-AS3	-2.565988
KIAA1908	3.4127824	LOC100505495	-3.384433
XLOC_008466	3.2270826	MGC27382	-4.104419
PRSS30P	3.149014	LOC400550	-4.5237017
LINC00665	2.945509	XLOC_001412	-4.6995843
RGMB-AS1	2.6495147	XLOC_l2_006399	-4.907096
HOTAIR	2.581743	RP11-165H20.1	-5.3639335

### Correlation between lncRNA DLX6-AS1 expression levels and clinical characteristics

**Figure 2:**
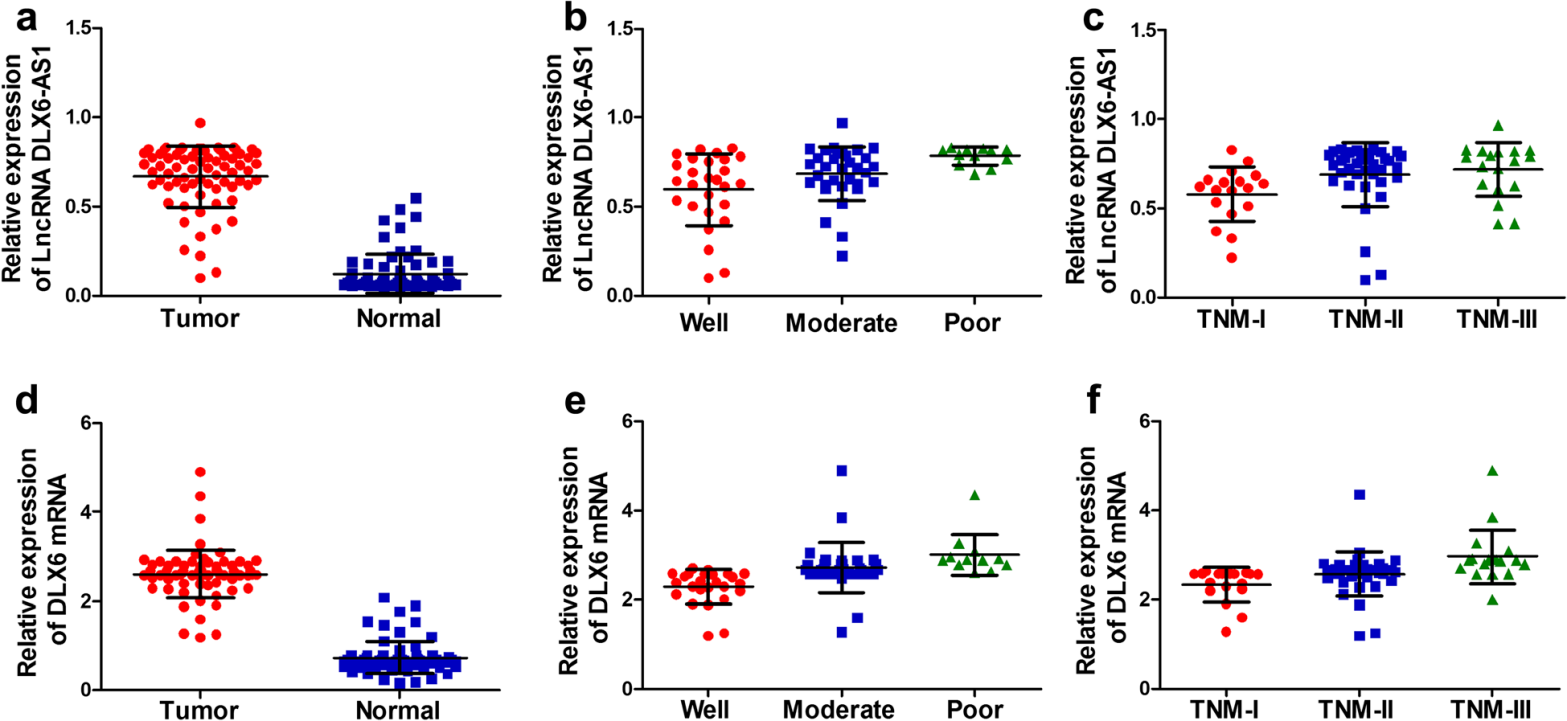
The expression levels of lncRNA DLX6-AS1 in lung adenocarcinoma tissues and adjacent normal lung tissues. **a** The expression levels of lncRNA DLX6-AS1 in paired LAC tissues and adjacent normal lung tissues were determined by qRT-PCR (2^−ΔCt^). Statistically significant differences were observed (*P< 0.05). **b** LncRNA DLX6-AS1 expression levels were significantly associated with histological differentiation (*P< 0.05). **c** The expression levels of lncRNA DLX6-AS1 were associated with TNM stage (*P< 0.05). **d** DLX6 mRNA levels in paired LAC tissues and adjacent normal lung tissues were determined by qRT-PCR(*P< 0.05). **e** DLX6 mRNA levels were significantly associated with histological differentiation (*P< 0.05). **f** DLX6 mRNA levels were associated with TNM stage (*P< 0.05). Tumor: tumor tissue; Normal: adjacent normal lung tissue.

GO annotation and KEGG pathway analysis indicated that aberrantly expressed of lncRNA DLX6-AS1 affected biological processes of lung adenocarcinoma. To validate the microarray data, we analyzed DLX6-AS1 expression in 72 paired LAC and adjacent normal lung tissues using qRT-PCR. We calculated the relative expression of lncRNA with the comparative cycle threshold (Ct) (2^−ΔCt^) method, and fold changes of ≥2 were designated as up-regulated. Up-regulation of DLX6-AS1 was detected in 83.33% (60/72) of LAC tissues compared with adjacent normal tissues, while 16.67% (12/72) displayed either down-regulation or unobvious alteration. DLX6-AS1 expression in LAC tissues was conclusively proved a higher level than that in paired adjacent normal lung tissues (Z = −7.273, *P* = 0.000, Figure 2a), while DLX6 mRNA showed a similar trend (Z = −7.374, *P* = 0.000, Figure 2d). Statistical analysis revealed that DLX6-AS1 and DLX6 mRNA expression levels were significantly associated with both histological differentiation (*P* = 0.004, Figure 2b; *P* = 0.000, Figure 2e) and TNM stage (*P* = 0.033, Figure 2c*; P* = 0.001, Figure 2f). No correlation was found with other clinicopathological features including age, gender, and lymph node metastasis (Table 2).

**Table 2:** **LncRNA DLX6-AS1 and DLX6 mRNA expression levels were associated with clinicopathological features of LAC Patients**

**Clinicopathological factor**	**n**	**LncRNA DLX6-AS1** **expression (2** ^**–**Δ**Ct**^ **)**		**DLX6 mRNA** **expression(2** ^**–**Δ**Ct**^ **)**	
		**Median ± SD**	***P-*** **Vale**	**Media ± SD**	***P*** **-Value**
Gender					
Male	35	0.6452±0.1905	0.249	2.5103±0.4123	0.149
Female	37	0.6926±0.1541		2.6941±0.6363	
Age (years)					
≥60	34	0.6762±0.1837	0.762	2.6426*±*0.6820	0.591
<60	38	0.6637±0.1655		2.5709±0.3863	
Differentiation					
Well	27	0.5965±0.2017	0.004*	2.2947±0.3855	0.000*
Moderate	33	0.6873±0.1510		2.7153*±*0.5559	
Poor	12	0.7852±0.4988		2.9984*±*0.4624	
Lymph node metastasis					
Positive	33	0.7078±0.1259	0.075	2.7230*±*0.5027	0.087
Negative	39	0.6373±0.2009		2.5047*±*0.5625	
TNM stage					
I	18	0.5806±0.1533	0.033*	2.3290±0.3853	0.001*
II	36	0.6897±0.1806		2.5641±0.4939	
III	18	0.7184±0.1512		2.9618±0.6028	

*Indicated statistical significance (*P*< 0.05).

### Down-regulation the expression of lncRNA DLX6-AS1 decreased DLX6 mRNA and protein levels

**Figure 3:**
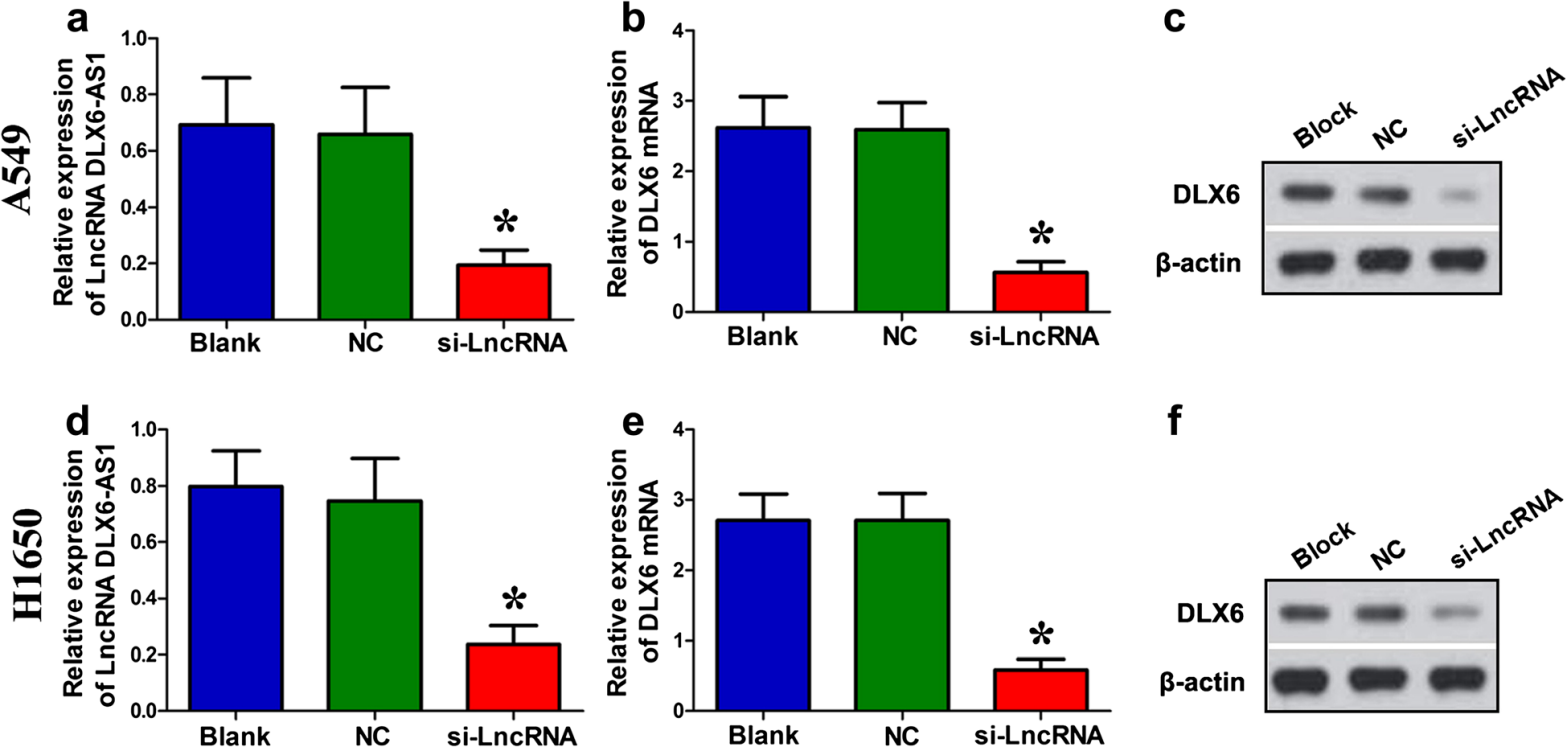
Down-regulation the expression of lncRNA DLX6-AS1 decreased the DLX6 protein levels. **a** Using qRT-PCR analysis in A549 cells, the expression levels of lncRNA DLX6-AS1 in si-LncRNA group were significantly lower, compared to NC and Blank groups (*P< 0.05). **b** DLX6 mRNA expression in si-LncRNA group was also lower than in the NC and Blank groups in A549 cells by qRT-PCR (*P< 0.05). **c** Western blotting analysis showed that DLX6 protein levels in si-LncRNA group were also lower than NC and Blank groups in A549 cells (*P< 0.05). β-actin was used as an endogenous reference. **d** qRT-PCR analysis verified that the expression level of lncRNA DLX6-AS1 in si-LncRNA group was significantly lower, compared to NC and Blank groups in H1650 cells (*P< 0.05). **e** DLX6 mRNA expression in si-LncRNA group was also lower than in NC and Blank groups in H1650 cells by qRT-PCR (*P< 0.05). **f** Western blotting analysis showed that DLX6 protein levels in si-LncRNA group were also lower than in the NC and Blank groups in H1650 cells (*P< 0.05). si-LncRNA group: cells transfected with siRNA targeting lncRNA DLX6-AS1; NC group: cells transfected with negative control siRNA; Blank group: non-transfected cells.

In most samples, the expression levels of lncRNA DLX6-AS1 were higher in tumor tissues than adjacent normal tissues. Thus, we speculated that DLX6-AS1 was an oncogene and knockdown of DLX6-AS1 decreased DLX6 expression. To test it, we investigated the changes of DLX6 mRNA and protein levels after A549 and H1650 cells were transfected with siRNA targeting lncRNA DLX6-AS1 (si-LncRNA groups), negative control (NC groups) blank. Blank group were set as well. qRT-PCR analysis revealed that lncRNA DLX6-AS1 expression levels in si-LncRNA groups were significantly lower, compared to NC and Blank groups (Figure 3a: A549,χ^2^ = 19.481, *P* = 0.000; Figure 3d: H1650, χ^2^ = 19.564, *P* = 0.001 ). qRT-PCR and Western blotting analysis showed that DLX6 mRNA and protein levels were significantly lower in si-lncRNA groups than in NC and Blank groups (Figure 3b, c, e, and f).

## Discussion

In recent years, genome-wide surveys have revealed that there are over 3000 human lncRNAs longer than 200 nt, but less than 1% have been characterized [33,34]. Although only a minority have been characterized in details, recent studies have indicated that lncRNAs were key players in gene regulatory processes and could influence both normal and transformed cellular function [35,36]. Several studies have further demonstrated that lncRNAs were efficiently regulated during cellular development in response to diverse signalings, and dysregulation of lncRNAs might also affect epigenetic information and provide advantages for cellular growth, resulting in progressive and uncontrolled tumor growth [17,29,36,37]. Thus, identification of tumor associated lncRNAs, which might be novel therapeutic targets, is critical for recognizing the roles of lncRNAs in tumorigenesis [38]. Based on microarray data analysis, our attention was focused on the lncRNA DLX6-AS1.

DLX gene family contains a homeobox related to Drosophila distal-less (Dll), a gene expressed in the head and limbs of developmental fruit fly. DLX genes, the vertebrate homologues of Dll, comprise a family of homeobox transcription factors involved in cell differentiation and morphogenesis. In humans, six distal-less homeobox (DLX) genes exist, represented by three bi-gene clusters, DLX1/DLX2, DLX5/DLX6, and DLX3/DLX4. Deregulation of DLX genes, including DLX4 and DLX5, was found in human solid tumors and hematologic malignancies, which indicated that DLX played an important role in tumor growth and progression [39-44]. So far, the functions of DLX6 haven’t been reported in related researches, not to mention its expression level and working mechanisms in lung cancer.

In this study, microarray analysis suggested that the expression level of lncRNA DLX6-AS1 was higher in three LAC tissues than that in the paired adjacent normal lung tissues. qRT-PCR was performed to investigate lncRNA DLX6-AS1 expression levels in 72 LAC patients who hadn’t received radiotherapy or chenmotherapy before. Correlation of lncRNA DLX6-AS1 expression levels and clinicopathological characteristics was then analyzed. Results showed that DLX6-AS1 expression was increased in LAC tissues compared with adjacent non-tumor tissues, which indicated its possible participation in carcinogenesis. Besides, DLX6-AS1 expression levels showed close correlation with LAC histological grade and TNM stage. High DLX6-AS1 expression levels were more likely to be detected in tumors with advanced histological grade, and TNM III, suggesting that DLX6-AS1 might participate in LAC invasion and metastasis. Taken together all the evidences, DLX6-AS1 was proposed to play a vital role in LAC carcinogenesis and progression. After transfection with siRNA of lncRNA DLX6-AS1, qRT-PCR and Western blotting analysis discovered that DLX6 mRNA and protein expression were decreased in A549 and H1650 cells compared to the NC and Blank groups. Based on our research data, lncRNA DLX6-AS1 was probably a novel therapeutic target for LAC patients. But the detailed molecular mechanism remains unclear. Our further studies will put more efforts on exploring the potential roles of lncRNA DLX6-AS1 in LAC development.

## Conclusions

Microarray analysis identified that lncRNA DLX6-AS1 was up-regulated in LAC tissues. High DLX6-AS1 expression levels were significantly associated with both histological differentiation and TNM stage. Down-regulation of lncRNA DLX6-AS1 expression decreased the DLX6 mRNA and protein levels.

## Materials and methods

### Patient samples collection

72 paired LAC tissues and adjacent normal lung tissues (located more than 5 cm away from the tumors) were obtained from patients who underwent primary surgical resections between March 2012 and January 2014 at the First Affiliated Hospital of Zhengzhou University. None of the patients had received preoperative adjuvant therapy. Resected tissue samples were immediately frozen in liquid nitrogen and stored at-80°C before RNA extraction. All the diagnoses of LAC and adjacent normal lung tissues were histopathologically confirmed. Prior informed consents and approval from the ethics committee of Zhengzhou University were obtained for the use of clinical samples for research purposes.

### Microarray assay

Paired cancer tissues and adjacent normal tissues from three patients were randomly pooled and hybridized to chips. LncRNA microarray assays were performed by Biotechnology Corporation (Shanghai, China).Total RNA was amplified and labeled by Low Input Quick Amp Labeling Kit, One-Color (Agilent technologies), following the manufacturer’s instructions. Labeled cDNA were purified by RNeasy mini kit (QIAGEN, Germany). After 17 hours’ hybridization, slides were washed with Gene Expression Wash Buffer Kit (Agilent technologies), following the manufacturer’s instructions. Slides were scanned by Agilent Microarray Scanner (Agilent technologies) with default settings. Data were extracted with Feature Extraction software 10.7 (Agilent technologies).

### RNA extraction and quantitative real-time fluorescent

To further certify the generality of lncRNAs expression pattern, specimens from 72 patients were used to perform qRT-PCR assay. Total RNA was extracted from frozen specimens using TRIzol reagent (Invitrogen, USA) according to the manufacturer’s protocol. qRT-PCR was performed using an ABI 7500 thermal cycler, according to the manufacturer’s recommendations. Each qRT-PCR experiment was repeated three times. The primers selected as the followings: lncRNA DLX6-AS1, forward, 5’-AGTTTCTCTCTAGATTGCCTT-3’; reverse, 5’-ATTGACATGTTAGTGCCCTT -3’. DLX6, forward, 5’-TCCACACCAGGACACGATGC-3’, reverse, 5’-CTTGCCACACTTATGAGCTCT-3’. GAPDH, forward, 5’-AGAGGCAGGGATGATGTTCTG -3’, reverse, 5’-GACTCATGACCACAGTCCATGC-3’. Relative expression of genes was calculated using the comparative cycle threshold (Ct) (2^−ΔCt^, ΔCt = Ct median lncRNA or mRNA-Ct median GAPDH) method with glyceraldehyde-3-phosphate dehydrogenase (GAPDH), as the endogenous control [45].

### Cell culture

Human lung adenocarcinoma cell lines A549 and H1650 were purchased from the Type Culture Collection of the Chinese Academy of Sciences (Shanghai, China). Cells were cultured in RPMI1640 (Gibco, USA) medium supplemented with 10% fetal bovine serum (FBS), 100 U/ml penicillin and 100 μg/ml streptomycin (Enpromise, China) in humidified air with 5% CO_2_ at 37°C.

### SiRNA transfection

Specific siRNAs targeting lncRNA DLX6-AS1 and the negative control siRNAs were designed and synthesized by GenePharma (Shanghai, China). Cells were plated in 6-well plates in antibiotic-free growth medium supplemented with 10% FBS and cultured until 50-70% confluent overnight. The siRNAs were transfected into cultured cells using Lipofectamine 2000 (Invitrogen, USA) according to the manufacturer’s instructions. Forty-eight hours after transfection, cells were harvested for qRT-PCR or Western blotting analysis.

### Western blotting

Cells were washed twice with PBS solution, and then were lysed with RIPA Lysis Buffer (Beyotime, China) containing phenylmethanesulfonylfluoride (PMSF). Protein concentrations were determined with Pierce BCA Protein Assay Kit (Beyotime, Haimen, China). Proteins were subjected to 12% sodium dodecyl sulfate polyacrylamide gel electrophoresis (SDS-PAGE) and transferred onto PVDF membranes. Membranes were blocked for 1 h in phosphate-buffered saline/Tween-20 containing 5% non-fat milk. The membranes were incubated overnight at 4°C with 1:500 dilutions of the primary antibodies (rabbit anti-human DLX6 antibody, 1:500, Santa Cruz Biotechnology). Following extensive washing with TBST, secondary antibodies were incubated at room temperature for 1 h (HRP labeled goat anti-rabbit IgG, 1:1000, Santa Cruz Biotechnology). Membranes were washed with Tween 20-PBS four times (15 min each) at room temperature. Signals were determined using a chemiluminescence detection kit (Amersham Pharmacia Biotech, Piscataway, NJ). Protein levels were normalized to β-actin.

### Statistical analysis

Statistical analysis was conducted with the assistance of SPSS 17.0 software. All data were expressed as means ± standard deviation (SD). The differences between paired samples were analyzed using Wilcoxon signed-rank test. The differences between diverse independent groups were analyzed using Kruskal-Wallis test. For multiple comparisons, Tamhane’T2 (M) was used in the comparison of the parental and control vector groups. *P*< 0.05 was considered to be statistically significant.
